# Serum and cerebrospinal fluid biomarker profiles in acute SARS-CoV-2-associated neurological syndromes

**DOI:** 10.1093/braincomms/fcab099

**Published:** 2021-05-12

**Authors:** Ross W Paterson, Laura A Benjamin, Puja R Mehta, Rachel L Brown, Dilan Athauda, Nicholas J Ashton, Claire A Leckey, Oliver J Ziff, Judith Heaney, Amanda J Heslegrave, Andrea L Benedet, Kaj Blennow, Anna M Checkley, Catherine F Houlihan, Catherine J Mummery, Michael P Lunn, Hadi Manji, Michael S Zandi, Stephen Keddie, Michael Chou, Deepthi Vinayan Changaradil, Tom Solomon, Ashvini Keshavan, Suzanne Barker, Hans Rolf Jäger, Francesco Carletti, Robert Simister, David J Werring, Moira J Spyer, Eleni Nastouli, Serge Gauthier, Pedro Rosa-Neto, The UCLH Queen Square COVID-19 Biomarker Study group, Mohammed R Ashraghi, Rubika Balendra, Guru Kumar, Soon Tjin Lim, Nicki Longley, Kiran Samra, Arvind Chandratheva, Hannah Cohen, Maria Efthymiou, Laura Zambreanu, Alexander Foulkes, Henrik Zetterberg, Jonathan M Schott

**Affiliations:** 1 University College London, Queen Square Institute of Neurology, London, UK; 2 National Hospital for Neurology and Neurosurgery, University College London Hospitals NHS Foundation Trust, Queen Square, London, UK; 3 Darent Valley Hospital, Dartford, Kent, UK; 4 UK Dementia Research Institute, London, UK; 5 UCL Institute of Neurology, Stroke Research Centre, Russell Square House, London, UK; 6 University of Liverpool, Brain Infections Group, Liverpool, Merseyside, UK; 7 Laboratory of Molecular and Cell Biology, UCL, Gower St, Kings Cross, London, UK; 8 University College London Institute of Immunity and Transplantation, London, UK; 9 Francis Crick Institute, London, UK; 10 Department of Psychiatry and Neurochemistry, Institute of Neuroscience & Physiology, the Sahlgrenska Academy at the University of Gothenburg, Mölndal, Sweden; 11 King’s College London, Institute of Psychiatry, Psychology & Neuroscience, Maurice Wohl Clinical Neuroscience Institute, London, UK; 12 Advanced Pathogens Diagnostic Unit, University College London Hospitals NHS foundation trust, London, UK; 13 Hospital for Tropical Diseases, University College Hospitals London, London, UK; 14 Department of infection and immunity, University College London, London, UK; 15 Department of clinical virology, University College London Hospitals NHS foundation trust, London, UK; 16 National Institute for Health Research Health Protection Research Unit in Emerging and Zoonotic Infections, Institute of Infection, Veterinary and Ecological Sciences, University of Liverpool, Liverpool, UK; 17 Walton Centre NHS Foundation Trust, Liverpool, UK; 18 Translational Neuroimaging Laboratory, McGill University Research Centre for Studies in Aging, Montreal, Canada; 19 Alzheimer’s Disease Research Unit, Douglas Research Institute, Le Centre intégré universitaire de santé et de services sociaux (CIUSSS) de l'Ouest-de-l'Île-de-Montréal, Canada; 20 Department of Neurology and Neurosurgery, Psychiatry and Pharmacology and Therapeutics, McGill University, Montreal, Canada

**Keywords:** COVID-19, NFL, encephalitis, ADEM

## Abstract

Preliminary pathological and biomarker data suggest that SARS-CoV-2 infection can damage the nervous system. To understand what, where and how damage occurs, we collected serum and CSF from patients with COVID-19 and characterised neurological syndromes involving the peripheral and central nervous system (n = 34). We measured biomarkers of neuronal damage and neuroinflammation, and compared these with non-neurological control groups, which included patients with (n = 94) and without (n = 24) COVID-19. We detected increased concentrations of neurofilament light, a dynamic biomarker of neuronal damage, in the CSF of those with central nervous system inflammation (encephalitis and acute disseminated encephalomyelitis) (14800pg/mL [400, 32400]), compared to those with encephalopathy (1410pg/mL [756, 1446], peripheral syndromes (GBS) (740pg/mL [507, 881]) and controls (872pg/mL [654,1200]). Serum neurofilament light levels were elevated across patients hospitalised with COVID-19, irrespective of neurological manifestations. There was not the usual close correlation between CSF and serum neurofilament light, suggesting serum neurofilament light elevation in the non-neurological patients may reflect peripheral nerve damage in response to severe illness. We did not find significantly elevated levels of serum neurofilament light in community cases of COVID-19 arguing against significant neurological damage. Glial fibrillary acidic protein, a marker of astrocytic activation, was not elevated in the CSF or serum of any group, suggesting astrocytic activation is not a major mediator of neuronal damage in COVID-19.

Abbreviations: ADEM: acute demyelinating encephalomyelitis; CNS: central nervous system; CRP: C-reactive protein; CSF: cerebrospinal fluid; CT: computed tomography; CXR: chest X-ray; GFAP: glial fibrillary acidic protein; ICU: intensive care unit; IV: intravenous; MRI: magnetic resonance imaging; N/A: not applicable; NFL: neurofilament light; NR: no result; PCR: polymerase chain reaction; PNS: peripheral nervous system.

## Introduction

SARS-CoV-2, causing COVID-19, may damage the nervous system. Following an initial report,[1] several case series have reported neurological and neuropsychiatric syndromes associated with SARS-CoV-2.[2,3] Neuroimaging and neuropathological studies have demonstrated a range of abnormalities,[4] including multi-territory infarcts, cerebral microbleeds, and white matter lesions.[5] The pathogenic mechanisms are unclear but may include direct viral CNS infection, para- and post-infectious inflammation, hypercoagulability and the consequences of critical illness.[3]

Biomarkers of infection, neuronal injury, and astrocytic activation are a means of understanding brain pathobiology *in vivo*. In particular, neurofilament light chain protein (NfL) is a dynamic marker of active neuronal damage.[6,7] Glial fibrillary acidic protein (GFAp), highly expressed in astrocytes, is a biomarker of astrocytic activation/injury[8] and Neopterin is a marker of immune activation with prognostic value.[9] Sampling biomarkers in different biological compartments (CSF and blood) permits further anatomical localisation.

Recent blood biomarker studies reported elevated concentrations of plasma GFAp and NfL, in severe but neurologically uncharacterised COVID-19 cases compared to controls.[10] As yet, it is unclear whether neuronal damage is restricted to SARS-CoV-2-associated neurological syndromes, and if so which clinical syndromes, or whether neuronal damage occurs as part of the syndrome of COVID-19 more generally.

Sampling CSF gives a much more direct approach to measuring biomarkers of CNS injury than sampling blood; typically they are 10-100 fold higher. As yet, few case reports/series of CSF in acute SARS-CoV-2 infection are available.

The aims of this study were to (a) evaluate if SARS-CoV-2 damages the nervous system in individuals who manifest clinical neurological syndromes; (b) compare neuronal injury markers in individuals with SARS-CoV-2 with and without clinically apparent neurological involvement; and (c) compare biomarker signatures in CSF and blood.

## Materials and Methods

### Subjects

We prospectively recruited patients presenting to our hospitals between March and June 2020 with probable/confirmed COVID-19.[11,12] Confirmed cases were diagnosed by polymerase chain reaction test for SARS-CoV-2 on nasopharyngeal swab or positive IgG SARS-CoV-2. Participants had new neurological signs or symptoms according to standardised definitions[11] within 40 days of respiratory or systemic COVID-19 symptoms (COVID-neurological). Additional blood and CSF samples were collected alongside samples collected as part of standard clinical care with written informed consent. Serum biomarkers of neuronal damage and astrocytic activation (*i.e.*, neurofilament light [NfL], total tau [T-tau], phosphorylated tau [P-tau] glial fibrillary astrocytic protein [GFAp]), were compared with levels in two separate control groups. The first, individuals who presented to University

College Hospital with probable/confirmed COVID-19 between March and June 2020 without neurological signs/symptoms or history of major neurological disease (COVID hospitalised). The second cohort comprised individuals with positive SARS-CoV-2 PCR who did not require hospital admission (COVID non-hospitalised), and had serum samples collected in a previously described study.[13] CSF biomarkers were compared to neurologically healthy amyloid, tau and translocator protein (TSPO) PET-negative controls (n=24) from a healthy ageing study, collected prior to the COVID-19 pandemic (non-COVID controls).

We further classified COVID-neurological cases as (1) 'central': encephalitis; encephalopathy; acute disseminated encephalomyelitis (ADEM) or stroke; (2) 'peripheral': Guillain-Barré syndrome (GBS) or (3) other according to clinical consensus criteria as previously described.[2]

Clinical data were extracted retrospectively and blindly from electronic records (EPIC, Madison, WI, USA). Clinical outcomes were determined based on electronic notes review January 2021 based on the most recent clinical correspondence, as previously described.[2] All individuals have been followed up after discharge for between 1 and 9 months. Potential confounders and effect modifiers including age, sex, full blood count, C-reactive protein, D-dimer, minimum percentage oxygen saturation, timing of symptom onset to biomarker sampling, and co-morbidities were determined. The earliest available blood result was taken, and lowest oxygen saturation was recorded.

## Consent

Subjects consent was obtained according to the Declaration of Helsinki. This study was approved by the Queen Square ethics committee and the Canada Research Ethics Board.

**Data Availability**

The data that support the findings of this study are available from the corresponding author, upon reasonable request.

**Biomarker collection, analysis and interpretation**

Serum NfL and GFAp concentrations in the COVID-neurological, COVID hospitalised and non-COVID control groups were measured by Single molecule array (Simoa),[14] using a single batch of reagents; intra-assay coefficients of variation were <10% for both analytes. COVID non-hospitalised serum was analysed using the Simoa NF-light assay (Quanterix, Billerica, MA) at a different laboratory; 23 independent samples were analysed across both sites and a correction factor used for standardisation. CSF NfL and GFAp concentrations were measured using in house ELISAs, as previously described in detail.[15,16] T-tau, P-tau181, amyloid β 1-40 and 1-42 (Aβ40 and Aβ42, respectively) were analysed by Lumipulse assays (Fujirebio, Ghent, Belgium). All measurements, except serum NfL, were performed using a single batch of reagents (intra-assay coefficients of variation were <10%), on randomized samples without information on clinical data. Positive brain amyloid status was defined as Aβ42/Aβ40 ratio <0.065 based on local clinical cut points, to determine whether amyloidosis could be a confounding factor in neurodegenerative biomarker interpretation.

**Imaging Analysis**

Two radiologists, blinded to biomarker data, assessed scans using standardised visual rating scales to quantify ischaemic stroke (ASPECTS),[17] pc-ASPECTS,[18] and white matter hyperintensities (ARWMC).[19] ARWMC scores were categorized as normal (0), intermediate (1-9) or severe (≥10). Discrepancies were resolved by consensus.

## Statistical analysis

Continuous variables were summarized using means and medians and compared using Student's independent-samples t-test or Mann-Whitney U test as appropriate. Categorical data were represented as percentages and compared using chi-squared test. The CSF and serum measures required common logarithm transformation to normalize the distributions for subsequent analysis so that assumptions would be met. Linear correlations between common logarithm-transformed CSF/serum NfL and other markers, were analysed by Pearson's product moment correlation, after transforming them using the common logarithm. The strength and direction of relationships was measured using simple linear regression, with examination of residuals to ensure fulfilment of linear regression assumptions. Individuals with missing data were excluded from that analysis. All statistical analyses and graphs were generated using Stata 14 (College Station, TX, USA) and Prism 8.3.1 (GraphPad, La Jolla, California, USA); $p<0.05$ was considered significant. We report medians / interquartile range.

## Results

### *Participant demographics and clinical features*

A total of 152 individuals were included in this study (Fig. 1). Thirty-four COVID-neurological cases (70% laboratory confirmed; 15% probable, 15% possible), 51 COVID hospitalised (78%

laboratory confirmed, 16% probable, 6% possible), 43 COVID non-hospitalised (100% laboratory confirmed) and 24 non-COVID controls were included. The COVID-neurological group consisted of central nervous system (CNS) (n=21: encephalopathy [n=8; 6/8 with CSF]; encephalitis [n=3; 3/3 with CSF]; ADEM [n=3; 2/3 with CSF]; stroke [n=7; no CSF], PNS (n=10; 9/10 with CSF) and other syndromes (n=3, 2/3 with CSF).

**Figure 1** here

Comparing the COVID-19 groups, the COVID-neurological group was younger than either the COVID hospitalised or the non-COVID control groups (Table 1). There were more males in the COVID-neurological group compared to the COVID non-hospitalised or non-COVID control groups. There were significantly more non-white individuals in the COVID hospitalised group (p=0.038). Further, CRP (p<0.001) and lymphocyte count (p=0.002) were significantly higher, while oxygen saturation (p=0.001) and haemoglobin (p=0.001) were significantly lower in the COVID hospitalised than the COVID-neurological group (*Table 1*). ~80% of the COVID hospitalised group required intensive care support, compared with only ~15% of the COVID-neurological group. 66% of the COVID CNS neurological group made a complete recovery.

**Table 1** here

***Standard clinical CSF markers of infection/inflammation***

In the COVID-neurological group, CSF samples were acellular (<5 white cells[WC]/uL) in all CSF except for a case of encephalitis (95 WC/uL) and a case of GBS (12 WC/uL). CSF protein was normal in all cases (<0.5g/dL) except in two cases of GBS (0.6 g/dL and 1.2 g/dL) and in one individual with encephalopathy (1.0 g/dL). All CSF samples were SARS-CoV-2 PCR-negative.

***Biomarkers of neuronal damage***

*Neurofilament light in CSF and serum*

We first determined whether CSF and serum NfL were correlated, in the two groups of subjects that had CSF available. In the COVID-neurological group CSF and serum NfL concentrations were not correlated ($r^2$=0.12, p=0.63), while in the non-COVID control group they were ($r^2$=0.63, p=0.002) (Fig. 2). We then compared CSF NfL between different neurological clinical presentations and controls. Median NfL was higher in the COVID-neurological group with CNS presentations [1510pg/mL (IQR; 857,14800)] compared to non-COVID controls [872pg/mL (654,1200)]; p=0.0477. Whereas, there were no differences between CSF NfL non-COVID controls compared to PNS cases [739pg/mL(IQR; 507,881)]: p=0.5083. To illustrate which clinical syndromes were driving this trend we provide boxplots of CSF NfL concentration by clinical syndrome (Fig. 2) which show highest levels of CSF NfL in ADEM followed by encephalitis groups. There was no significant difference between CSF NfL in the CNS and PNS groups (p=0.112). Two individuals had missing CSF values.

Serum NfL levels were elevated in COVID CNS neurological syndromes [51.0 pg/mL (IQR; 20.7,187)], COVID PNS neurological syndromes [122(25.7,293)] and COVID hospitalised [66.3pg/mL (IQR; 28.2,171.0)] p=0.001, compared to COVID non-hospitalised [6.7pg/mL (5.5,8.6)] and non-COVID controls [16.7pg/mL (11.6,24.5)] p=0.001. Six individuals had missing values.

*Glial Fibrillary Protein in CSF and Serum*

CSF and serum GFAp were not correlated in either the COVID-neurological group ($R^2$ 0.19, p=0.42) or the Non-COVID controls ($R^2$ 0.19, p=0.39). CSF GFAp concentrations were lower in COVID CNS neurological syndromes [3753pg/mL(517, 7660)] compared to non-COVID controls [9917.0pg/mL (8160.0,13600)]: p=0.002. CSF GFAp concentrations were also lower in COVID PNS neurological syndromes [2234.0pg/mL (2060, 6440)] compared to non-COVID controls (p=0.003). There was no significant difference in GFAp between CNS and PNS cases in CSF (p=0.804). One individual had missing values.

Serum GFAp was higher in COVID CNS neurological conditions [112.0pg/mL (53.9,402)], COVID PNS cases [110pg/mL (77.2,251.2)] and COVID hospitalised cases [125.8pg/mL (90.0,171.0)] compared to COVID non-hospitalised cases. (p=0.0171) Five individuals had missing values.

*Biomarkers of neuronal damage: exploring correlations with age, disease severity, hypoxia and BMI*

We explored the relationship between serum neuronal injury biomarkers and age, time from COVID-19 infection to sampling, disease severity (CRP) and hypoxia across all individuals with COVID-19. No association was observed between serum NfL and age (R2=0.0 p=0.886), CRP (R2=0.01, p=0.472), hypoxia (R2=0.03, p=0.122). There was a weak association between timing of blood sampling and serum NfL ($R^2$=0.07, p=0.027). There were no differences in timing of blood sampling between the COVID neurological and COVID hospitalised groups (p=0.222). No association was observed between serum GFAp and age ($R^2$=0.03, p=0.094), time from COVID-19 infection to blood sampling ($R^2$=0.04, p=0.108), CRP ($R^2$=0.04, p=0.092) or lowest percentage oxygen saturation ($R^2$=0.00, p=0.756).

*Correlating biomarkers of neuronal damage and brain imaging*

Brain imaging was available for twenty-six individuals in the COVID-neurological group (MRI, n=21; CT, n=5). Alberta stroke program early CT score (ASPECTS), pcASPECT and age-related white matter changes (ARWMC) scores were calculated for each and microhaemorrhages were identified in three (anatomical localisation provided in Table 1). We explored associations between imaging abnormalities and neuronal damage biomarkers in the COVID-neurological group. There was a significant association between severe white matter disease (AMWRC) and serum NfL (p=0.024) (Fig. 3) but not serum GFAp (p=0.157). No association was observed between ASPECTS scores, serum NfL or serum GFAp.

*Correlating biomarkers of neuronal damage with clinical outcomes*

We explored associations between clinical outcome and biomarkers of neuronal damage in CSF and serum in individuals with COVID-neurological CNS syndromes. Raised CSF NfL concentrations were associated with incomplete recovery and death (Fig. 3), but numbers were small and did not reach statistical significance. We did not observe associations with either serum NfL or GFAp.

***CSF biomarkers of Amyloidosis***

To understand the relationship between Alzheimer's disease pathology and the neurological manifestations of acute SARS-CoV-2 infection, CSF Aβ42/40 ratio, T-tau and P-tau were measured and compared with local clinical cut-points. Five individuals in the COVID-neurological group had CSF biomarkers suggestive of amyloidosis (3/10 GBS and 2/8

encephalopathy). None of these individuals had CSF biomarker evidence of elevated T-tau or P-tau or NfL. Two individuals had missing values.

***CSF Total Tau and P-tau***

CSF T-tau was higher in COVID-neurological CNS cases [585 (IQR; 220, 1788)] compared to COVID-neurological PNS cases [127 (IQR; 98, 162)] P=0.009, but not significantly higher than non-COVID controls [289 (243,356)]; P= 0.7491.

CSF P-tau was not elevated in COVID-neurological CNS [585 (IQR; 220, 1788)], COVID-neurological PNS [127 (IQR; 98, 162)] or non-COVID control groups [33 (27.8,37.9)]. Two individuals had missing values.

**Discussion**

This is the largest prospective biomarker study of carefully clinically characterised patients with neurological manifestations of COVID-19. It includes controls with and without COVID-19, covering the full spectrum of disease severity. We tested biomarkers in two compartments: CSF, most sensitive to reflect CNS pathobiology, and blood, a downstream repository of CNS derived biomarkers, which will also contain biomarkers released from the peripheral nervous system and other organs. This has provided us with further insight into the localisation and mechanisms of neuronal damage in acute COVID-19 disease. As well as relating these biomarkers to clinical syndrome, we have also been able to correlate these fluid biomarkers with imaging, neurodegenerative pathological biomarkers and clinical outcomes.

One of the major findings of this study is that CSF NfL is higher in patients with COVID-19 related neurological diseases of the CNS, but more specifically it was highest in individuals with ADEM and encephalitis. NfL is a cytoskeletal protein, expressed mainly in large fibre myelinated axons[20] which is thought to be passively released into CSF in response to neurological insults including head trauma,[21] neuroinflammatory relapses in multiple sclerosis,[6] and a range of neurodegenerative diseases, reflecting disease intensity.[22] It is therefore not surprising to see significant elevation in CSF NfL in those with severe CNS syndromes causing significant neurological disability with imaging changes involving the white matter. Levels were higher than in many neurodegenerative diseases and stroke.[6] It is also not surprising to see CSF NfL linked with clinical outcome.

Conversely, it was reassuring to observe only modest, non-significant elevations in CSF NfL in COVID encephalopathy. Encephalopathy has emerged as a frequent complication of acute SARS-Cov2 infection and is associated with a poorer functional outcome, independent of respiratory disease severity.[23] The mechanism of disease is not clear. Few cases have SARS-CoV-2 RNA in CSF,[24] and current thinking is that encephalopathy may reflect microvascular change, endothelial activation and local inflammation.[25] NfL is elevated in many neurological conditions and correlates with the intensity of neurodegeneration. The lack of CSF NfL elevation in encephalopathy cases without white matter involvement on imaging provides some evidence to suggest that these cases do not have excess neurodegeneration implying no significant long-term neurological deficit.

It was unexpected to observe a loss of the normally very close correlation between CSF and serum NfL[6] in acute SARS-CoV-2 infection. It is not clear how NfL released from the CNS

passes into peripheral blood. In one study, elevated serum NfL started to be detected an hour after injury in concussed hockey players,[26] and following minimal surgical brain injury, NfL levels in these two biofluids follow each other closely.[27] A close correlation is typically seen between NfL in these two compartments in steady disease states.[28] In this study the loss of correlation is partly driven by peripheral nerve syndrome cases, where there is elevated serum NfL but normal concentrations of CSF NfL. This implies that NfL is released from peripheral nerves into blood, and does not freely pass back into CSF.

We also observed no correlation between serum and CSF NfL in CNS cases, with high CSF NfL not necessarily reflected in peripheral blood. One possible explanation is the timing of sampling; it may take longer than previously thought for NfL levels to reach a steady state in blood. *In vivo* studies of NfL kinetics are under way to understand the kinetics of NfL and this will be critical for NfL interpretation in blood. The poor correlation between serum and CSF has important implications for future biomarker studies in the acute setting, suggesting that serum NfL may not be a reliable marker of CNS disease, and that CSF may be required to reliably detect or track neuronal damage in acute COVID-19.

Like others, we also observed elevated serum NfL levels in the hospitalised COVID-19 cohort.[10,14] This group was composed of individuals with moderate to severe COVID-19 who did not have pre-existing neurological disease/ recognised neurological sequelae. Other biomarker studies have detected evidence of neuronal damage in those with moderate to severe COVID-19 without clinically-manifest neurological disease.[10,14] Pathological studies have shown a range of pathological changes ranging from microinfarcts[5,29]to relatively mild neuropathological changes,[30,31] with no evidence of encephalitis or vasculitis[32] leaving some doubt as to whether elevated neuronal damage markers are derived from brain. The majority (~80%) of the COVID hospitalised cohort were managed in intensive care settings, where the median admission for COVID-19 is ~10 days[33] we postulate that elevated serum NfL may be a consequence of prolonged immobility causing critical care neuromyopathy or a combination of peripheral and central neuronal damage and not necessarily a reflection of subclinical CNS disease. Future studies incorporating peripheral neurophysiology are needed to confirm this.

Low CSF Nfl in PNS COVID-19 cases implies that CNS neuronal damage is not evitable in acute SARS-CoV-2 infection. Further reassurance is provided by serum NfL concentration in the non-hospitalised COVID cohort. This cohort consisted of individuals with confirmed SARS-CoV-2 who were under regular surveillance during the first peak of the pandemic, but did not require admission to hospital. Acknowledging the caveats in serum NfL interpretation discussed above, serum NfL levels were extremely low in this group, making it unlikely that this cohort sustained significant CNS or PNS damage during the acute illness. Since this is a cross-sectional study with biomarker collection during the acute illness we cannot predict whether this will predispose to longer term neuronal damage.

This is the largest study to show that standard CSF markers of CNS infection/inflammation (white cell count and total protein) are not altered in SARS-CoV-2 infection and confirms the findings of a number of other smaller studies. We were not able to detect direct evidence of SARS-CoV-2 RNA by PCR in CSF. A small number of cases have demonstrated positive SARS-CoV-2 in CSF at low levels, including a small detailed case series which identified positive CSF

PCR in three individuals with encephalopathy/meningism.[24] A lack of consistent evidence of viral RNA in CSF across a wide range of neurological presentations suggests that direct CNS invasion is not the major mechanism for neurological manifestations.[32] Low/normal GFAp in serum and CSF argues against astrocytic activation. However, it is possible that GFAp will not detect all forms of astrocyte reactivity.[34] Elevated CSF GFAp was detected in another cohort of acute COVID-19 patients, although this trend was driven by high levels in critically unwell patients.[35]

In our cohort a slightly larger number of individuals than expected had CSF biomarker evidence of amyloidosis. Three of these individuals had GBS, an acute demyelinating disease of the peripheral nervous system. A causative association with CNS amyloid deposition is therefore unlikely. CSF biomarker evidence of amyloidosis was seen in isolation, without biomarker evidence of tau pathology (phosphorylated tau) or neurodegeneration (NfL). Based on this small samples size, we did not see evidence to suggest that COVID-neurological syndromes were more common in those with underlying AD pathology.

A strength of this study is that participants were prospectively recruited and clinically classified according to agreed case definitions. We also had access to radiological and clinical data that allowed us to adjust for disease severity. The hospitalised COVID group had more severe COVID-19 infections than the COVID-neurological group, based on higher CRP and lower oxygen saturations, but disease severity, timing of sampling or medical co-morbidities did not influence biomarker concentrations.

There are a number of limitations to this study. Numbers are still relatively small, even though favourable when compared to existing studies. We did not have access to a control group with a different infection, therefore we cannot say how specific these findings are to SARS-CoV-2 infection. Samples could not be collected according to usual strict pre-handling standard operating procedures due to the clinical context. We know from a variety of CSF handling studies that this is important for measuring amyloid concentration, but less important for tau, NfL and other less hydrophobic proteins.[36] The non-COVID controls were older than the COVID-neurological group, although as serum NfL rises with age this if anything strengthens our findings.

In conclusion, CNS biomarkers of neuronal injury are highest in ADEM and encephalitis, and correlated with white matter abnormalities on imaging and incomplete clinical recovery. There is only weak evidence for significant neuronal damage in COVID encephalopathy. CSF and serum NfL are not correlated in acute SARS-CoV-2 infection: blood measures of NfL may not be a reliable measure of CNS dysfunction in COVID-19, and where this is suspected CSF evaluation should be considered. In non-neurological, hospitalised COVID-19 patients, subclinical neuronal peripheral or central nervous system damage maybe occurring but needs further investigation.

**Acknowledgements**

We are grateful to Coraline Daeninck, Jane Douglas and Anne Parnell for administrative support in running this study.

**Funding**

This study was supported by a grant from the University College London Hospitals National Institute for Health Research Biomedical Research Centre. This work is supported by UCL Queen Square Biomedical Research Centre (BRC) and Moorfields BRC grants (#560441 and #557595). RWP is supported by an Alzheimer's Association Clinician Scientist Fellowship (AACSF-20-685780) and the UK Dementia Research Institute. LB is supported by Global Challenges Research Fund and a Wellcome Trust Fellowship (222102/Z/20/Z). AK is supported by a Wolfson Clinical Research fellowship and the Weston Brain Institute (UB170045). RB is supported by a Medical Research Council (MRC) Clinical Research Training Fellowship. KB is supported by the Swedish Research Council (#2017-00915), the Alzheimer Drug Discovery Foundation (ADDF), USA (#RDAPB-201809-2016615), the Swedish Alzheimer Foundation (#AF-742881), Hjärnfonden, Sweden (#FO2017-0243), the Swedish state under the agreement between the Swedish government and the County Councils, the ALF-agreement (#ALFGBG-715986), and European Union Joint Program for Neurodegenerative Disorders (JPND2019-466-236). HZ is a Wallenberg Scholar supported by grants from the Swedish Research Council (#2018-02532), the European Research Council (#681712), Swedish State Support for Clinical Research (#ALFGBG-720931), the Alzheimer Drug Discovery Foundation (ADDF), USA (#201809-2016862), and the UK Dementia Research Institute at UCL. The TRIAD cohort was funded by the Weston Brain Institute, Canadian Institutes of Health Research (CIHR) (FRN, 152985, PI:PR-N) and Fonds de Recherche du Québec – Santé (FRQS; Chercheur Boursier, PR-N and 2020-VICO-279314).

**Competing interests**

RB, DA, CL, AK, JH, AH, NJA, ML, MC, SK, OZ, DVC, SB, FC, HJ, RS, AMS, TSL, RB, KS, GK and DW report no conflicts of interest.

RWP is co-principal investigator of the Neurofilament Light consortium, an industry funded consortium, and has received honoraria from GE Healthcare, outside the scope of this study.

H Cohen reports, outside the submitted work, institutional research support and support to attend scientific meetings from Bayer Healthcare, with honoraria for lectures from Bayer Healthcare and consultancy fees from UCB Biopharma paid to University College London Hospitals Charity.

LB reports a research grant from GlaxoSmithKline, outside the submitted work.

HZ has served at scientific advisory boards for Denali, Roche Diagnostics, Wave, Samumed, Siemens Healthineers, Pinteon Therapeutics and CogRx, has given lectures in symposia sponsored by Fujirebio, Alzecure and Biogen, and is a co-founder of Brain Biomarker Solutions in Gothenburg AB (BBS), which is a part of the GU Ventures Incubator Program (outside submitted work). KB has served as a consultant or at advisory boards for Axon, Biogen, CogRx, Lilly, MagQu, Novartis and Roche Diagnostics, and is a co-founder of Brain Biomarker Solutions in Gothenburg AB (BBS), which is a part of the GU Ventures Incubator Program. JMS declares personal fees from Axon Neuroscience, Roche, Eli Lilly, General Electric Healthcare, Merck Sharp & Dohme, Oxford University Press, Biogen, and EU Horizon 2020 outside the submitted work. CJM has served on advisory boards to Biogen, Roche, IONIS, and WAVE. MSZ has received lecturing fees for Eisai and UCB Pharma. TS was an adviser to the

GlaxoSmithKline Ebola Vaccine programme, chaired a Siemens Diagnostics clinical advisory board, and advises the WHO Brain Health Unit Forum on Neurology and COVID-19.

**Appendix**

The UCLH Queen Square COVID-19 Biomarker Study group: Mohammed R Ashraghi, Rubika Balendra, Guru Kumar, Soon Tjin Lim, Nicki Longley, Kiran Samra, Arvind Chandratheva, Hannah Cohen, Maria Efthymiou, Laura Zambreanu, Alexander Foulkes

## Figure legends

### Figure 1. Cohort overview

Flowchart describing cohorts. PNS: peripheral nervous syndrome; CNS: central nervous system.

### Figure 2. NfL and GFAp: scatter plot graphs of CSF against serum and boxplots by clinical groups

1) Scatter plot graphs of serum NfL against CSF NfL for COVID-neurological cases and non-COVID controls (A) ; Scatter plot graphs of serum GFAp against CSF serum GFAp for COVID-neurological cases and non-COVID controls (B); 2) Boxplots of CSF NfL (C) and GFAp (E) for neurological groups; 3) Boxplots of serum NfL (D) and GFAp (F) for neurological group. Serum and CSF NfL and serum and CSF GFAp are $log^{10}$ transformed. CSF: cerebrospinal fluid; NfL: neurofilament light; GFAp: glial fibrillary astrocytic protein. * <0.05, ***P* ≤ .01, ****P* ≤ .001, *****P* ≤.000, ns – non-significant.

### Figure 3. Boxplots of CSF and serum NfL for CNS group, radiological subtype and clinical outcome

Boxplots of CSF and serum NfL for 1) CNS group (clinical subtype: encephalopathy; encephalitis; ADEM) and non-COVID controls; 2) radiological subtype; 3) clinical outcome. The boxplots show the median and interquartile range (lower Q1 and upper Q3 quartiles) and the whiskers represent the entire range. Serum and CSF NfL are $log^{10}$ transformed. CSF: cerebrospinal fluid; NfL; serum neurofilament light; CNS: central nervous system; ADEM:

acute disseminated encephalomyelitis; and Age related white matter scale category (ARWMC).

**Table 1:** Demographic information for the COVID-neurological, COVID hospitalised, COVID non-hospitalised and Non-COVID control groups.

| | **COVID-Neurological (N=34)** | **COVID Hospitalised (N=51)** | **COVID non-hospitalised (N=29)** | **Non COVID-19 controls (N=24)** | **p** |
|---|---|---|---|---|---|
| **Median Age (IQR), years** | 45.5 (38,58) | 57 (50,65) | 43 (38,52) | 68(66,71) | **0.000** |
| **Male Sex (%)** | 18 (60) | 39 (78) | 13 (45) | 5 (31) | **0.003** |
| **Ethnicity** | | | NA | NA | |
| Non-white, % | 14 (47) | 35 (70) | | | **0.038** |
| White, % | 16 (53) | 15 (30) | | | |
| **Lowest Oxygen Saturation (IQR), %** | 96 (93,97) | 87 (77,96) | **NA** | **NA** | **0.007** |
| Hypertension | 6 (20) | 17 (34) | NA | NA | 0.180 |
| Diabetes | 6 (20) | 12 (24) | NA | NA | 0.109 |
| Median BMI (IQR) | 25.1 (22.6,29.9) | 24.7 (21.5,29.6) | NA | NA | 0.781 |
| D-dimer, ug/L | 1305 (855,4210) | 3665 (1700,6430) | NA | NA | 0.070 |
| Haemoglobin, g/L | 133 (118,149) | 108 (89,129) | **NA** | **NA** | **0.001** |
| Lymphocyte $10^9$/L | 1.68 (1.14,2.35) | 0.87 (0.64,1.33) | **NA** | **NA** | **0.002** |
| Platelet $10^9$/L | 267 (204,369) | 240 (207,359) | NA | NA | 0.560 |
| Creatinine umol/L | 71 (67,91) | 76 (63,109) | NA | NA | 0.535 |
| CRP µg/mL | 19 (6,94) | 182 (65,310) | **NA** | **NA** | **<0.001** |
| Time from COVID-19 symptom onset to blood sampling (days) | 14 (10,30) | 25 (14,33) | NA | NA | 0.222 |
| **Radiological burden of microhaemorrhage** | | | | | |
| Lobar | 1 (3) | NA | NA | NA | |
| Deep | 1 (3) | | | | |
| Corpus Callosum | 1 (3) | | | | |

Significant p values (defined as <0.05) are in bold. IQR: interquartile range; BMI: body mass index; CRP: C-reactive protein; NA: not available.

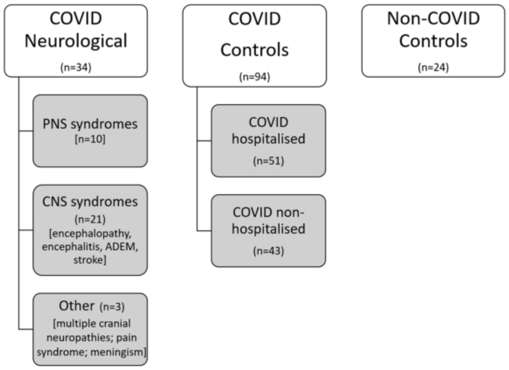

Figure 1. Flowchart describing cohorts. PNS: peripheral nervous syndrome; CNS: central nervous system.

169x95mm (300 x 300 DPI)

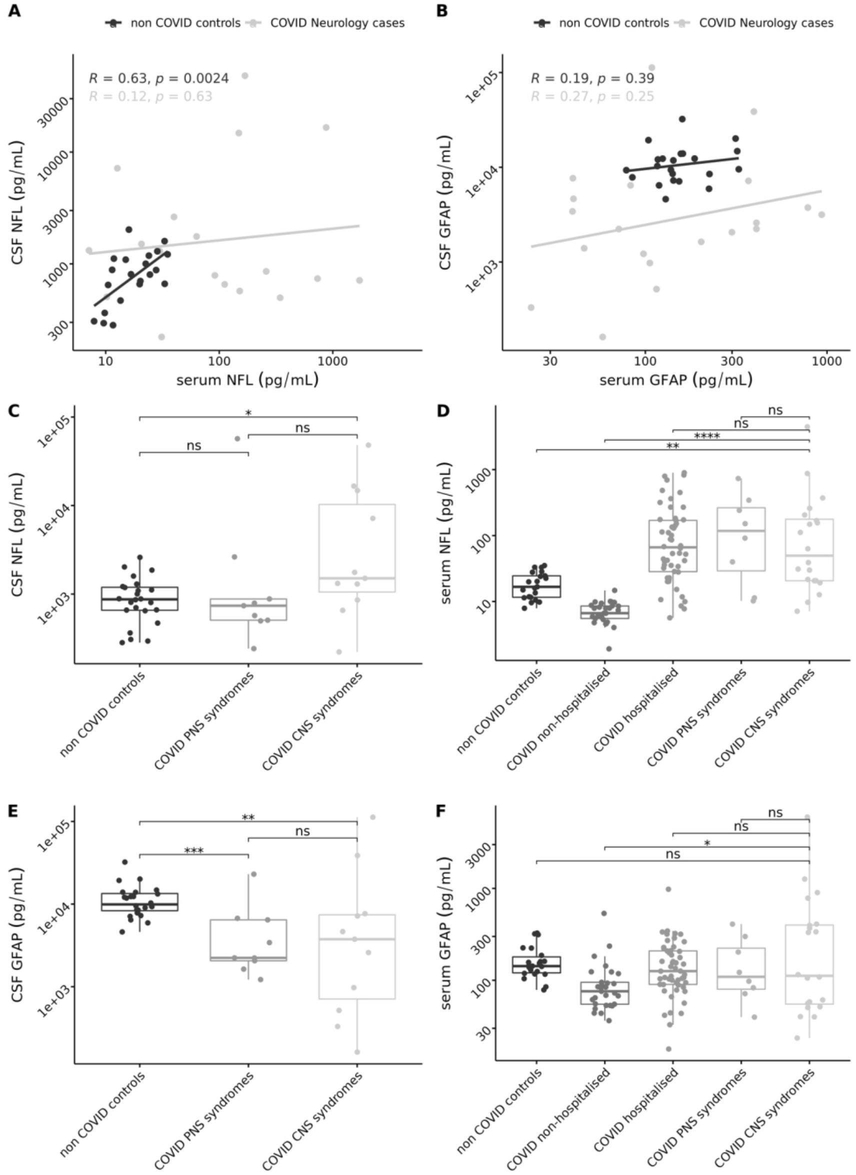

Figure 2. 1) Scatter plot graphs of serum NfL against CSF NfL for COVID-neurological cases and non-COVID controls (A) ; Scatter plot graphs of serum GFAp against CSF serum GFAp for COVID-neurological cases and non-COVID controls (B); 2) Boxplots of CSF NfL (C) and GFAp (E) for neurological groups; 3) Boxplots of serum NfL (D) and GFAp (F) for neurological group. Serum and CSF NfL and serum and CSF GFAp are log10 transformed. CSF: cerebrospinal fluid; NfL: neurofilament light; GFAp: glial fibrillary astrocytic protein. * <0.05, **P ≤ .01, ***P ≤ .001, ****P ≤.000, ns – non-significant.

127x177mm (300 x 300 DPI)

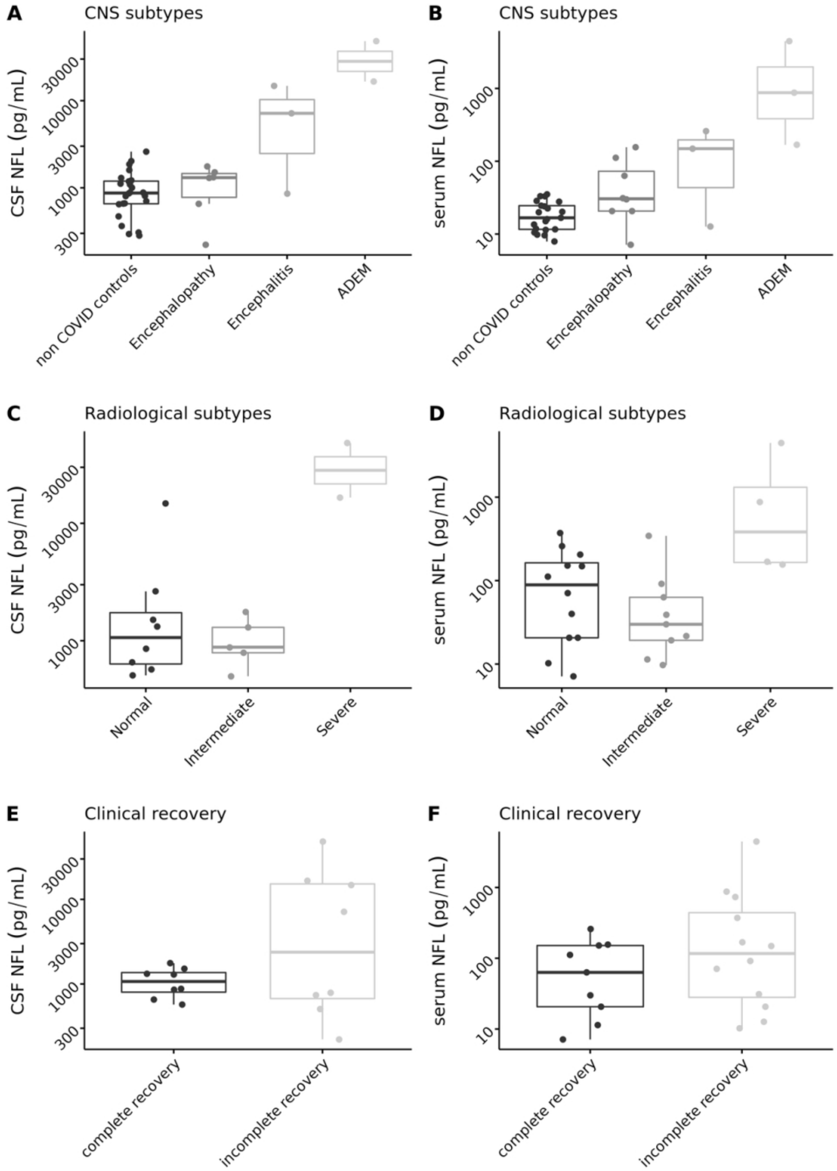

Figure 3. Boxplots of CSF and serum NfL for 1) CNS group (clinical subtype: encephalopathy; encephalitis; ADEM) and non-COVID controls; 2) radiological subtype; 3) clinical outcome. The boxplots show the median and interquartile range (lower Q1 and upper Q3 quartiles) and the whiskers represent the entire range. Serum and CSF NfL are log10 transformed. CSF: cerebrospinal fluid; NfL; serum neurofilament light; CNS: central nervous system; ADEM: acute disseminated encephalomyelitis; and Age related white matter scale category (ARWMC).

104x149mm (300 x 300 DPI)

Paterson, Benjamin et al. report elevation of CSF neurofilament light (NfL), reflecting central nervous system damage, in some but not all SARS-CoV-2 associated neurological syndromes. Increased NfL concentration was a common finding in hospitalised patients with COVID-19, likely predominantly reflecting peripheral neuronal damage. Non-hospitalised individuals did not have elevated NfL.

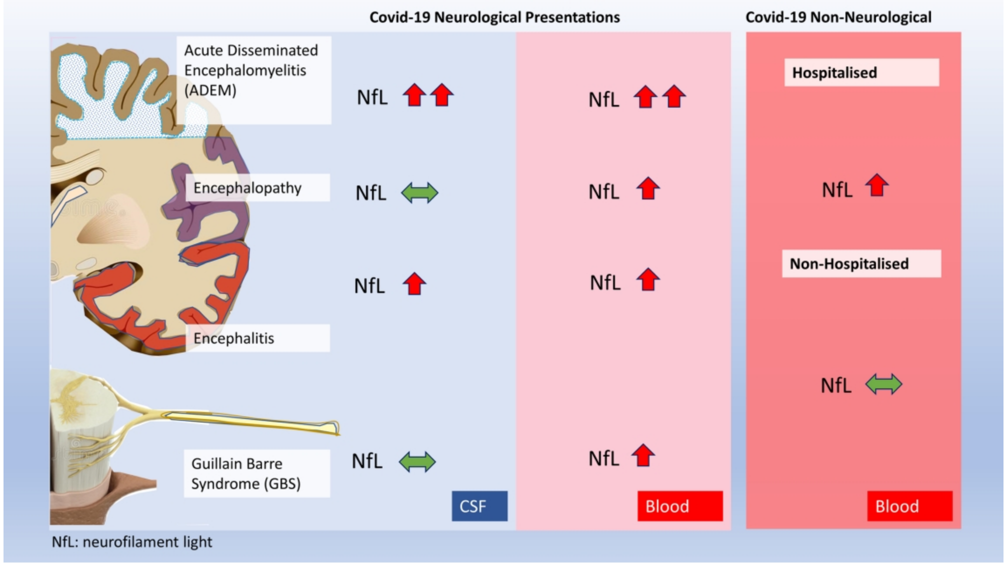

Graphical abstract

169x95mm (300 x 300 DPI)
